# Supplementary material for: Renal Toxicities in Cancer Patients Receiving Immune-Checkpoint Inhibitors: A Meta-Analysis
Source: J Clin Med. 2022 Jul 27;11(15):4373. doi: 10.3390/jcm11154373 (PMC9368813; doi:10.3390/jcm11154373)
Supplement: Supplementary file 1 [file jcm-11-04373-s001.zip › jcm-1779712-supplementary.pdf]

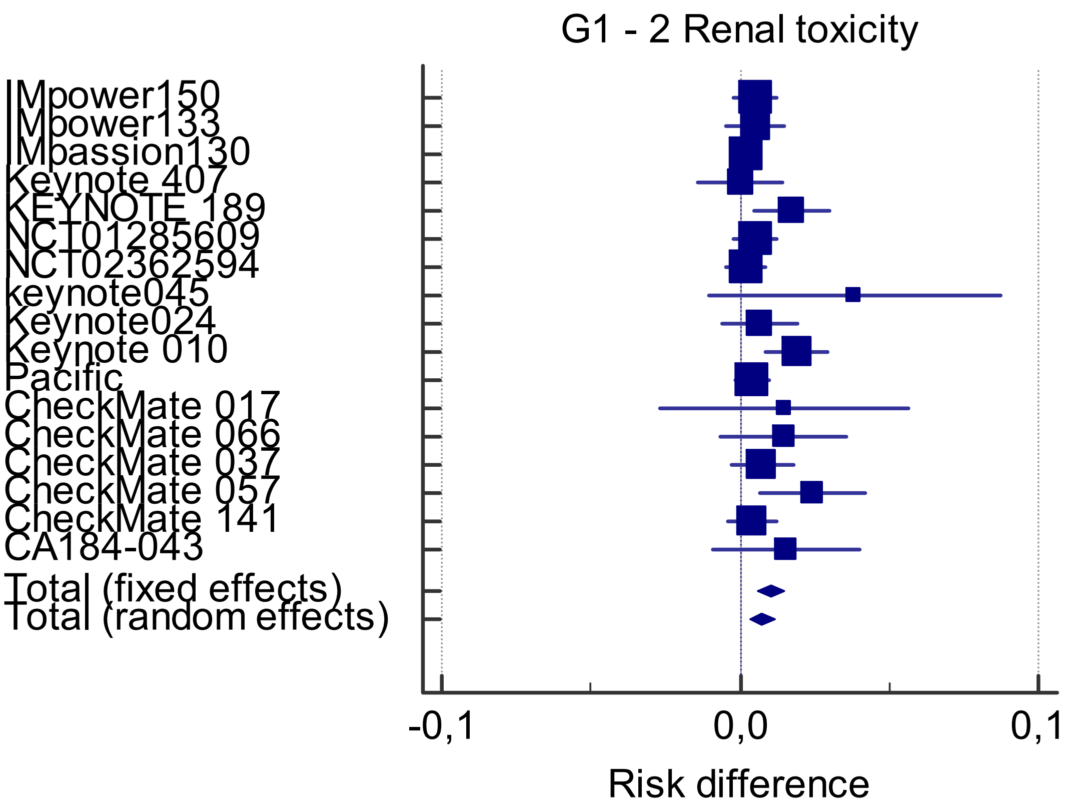


**Figure S1.** Grade 1 and Grade 2 renal toxicity.

**Table S1.** Grade 1 and Grade 2 renal toxicity.

| Study | Intervention | Controls | Risk Difference | 95% CI | z | P | Weight (%) | |
|---|---|---|---|---|---|---|---|---|
| | | | | | | | Fixed | Random |
| IMpower150 | 2/393 | 0/394 | 0.00509 | -0.00195 to 0.0121 | | | 9.43 | 8.65 |
| IMpower133 | 1/198 | 0/196 | 0.00505 | -0.00482 to 0.0149 | | | 4.79 | 6.92 |
| IMpassion130 | 1/452 | 0/438 | 0.00221 | -0.00212 to 0.00654 | | | 24.88 | 10.29 |
| Keynote 407 | 2/278 | 2/280 | 0.0000514 | -0.0139 to 0.0141 | | | 2.38 | 4.91 |
| KEYNOTE 189 | 7/405 | 0/202 | 0.0173 | 0.00459 to 0.0300 | | | 2.90 | 5.48 |
| NCT01285609 | 2/388 | 0/361 | 0.00515 | -0.00197 to 0.0123 | | | 9.19 | 8.59 |
| NCT02362594 | 2/509 | 1/502 | 0.00194 | -0.00475 to 0.00863 | | | 10.43 | 8.87 |
| keynote045 | 29/266 | 18/255 | 0.0384 | -0.0105 to 0.0873 | | | 0.20 | 0.66 |
| Keynote024 | 1/154 | 0/150 | 0.00649 | -0.00619 to 0.0192 | | | 2.90 | 5.48 |
| Keynote 010 | 13/682 | 0/309 | 0.0191 | 0.00880 to 0.0293 | | | 4.43 | 6.71 |
| Pacific | 2/475 | 0/234 | 0.00421 | -0.00161 to 0.0100 | | | 13.76 | 9.41 |
| CheckMate 017 | 5/131 | 3/129 | 0.0149 | -0.0270 to 0.0568 | | | 0.27 | 0.88 |
| CheckMate 066 | 4/206 | 1/205 | 0.0145 | -0.00658 to 0.0357 | | | 1.05 | 2.83 |
| CheckMate 037 | 2/268 | 0/102 | 0.00746 | -0.00284 to 0.0178 | | | 4.40 | 6.68 |
| CheckMate 057 | 7/287 | 0/268 | 0.0244 | 0.00654 to 0.0422 | | | 1.47 | 3.61 |
| CheckMate 141 | 1/236 | 0/111 | 0.00424 | -0.00405 to 0.0125 | | | 6.80 | 7.86 |
| CA184-043 | 16/393 | 10/396 | 0.0155 | -0.00945 to 0.0404 | | | 0.75 | 2.18 |
| Total (fixed effects) | 97/5721 | 35/4532 | 0.0105 | 0.00629 to 0.0146 | 4.913 | <0.001 | 100.00 | 100.00 |
| Total (random effects) | 97/5721 | 35/4532 | 0.00746 | 0.00337 to 0.0115 | 3.580 | <0.001 | 100.00 | 100.00 |

**Test for heterogeneity**

| | |
|---|---|
| Q | 42.9206 |
| DF | 16 |
| Significance level | P = 0.0003 |
| $I^2$ (inconsistency) | 62.72% |
| 95% CI for $I^2$ | 37.10 to 77.91 |

**Figure S2.** All grades renal toxicity.

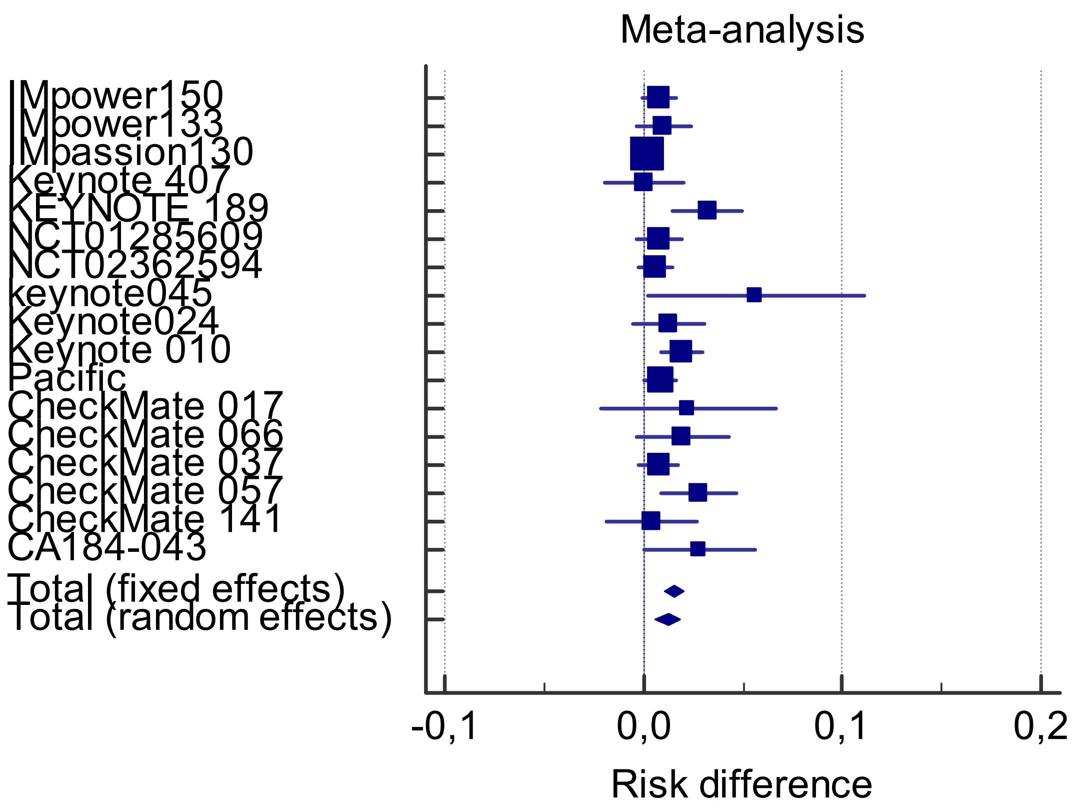

**Table S2.** All grades renal toxicity.

| Study | Intervention | Controls | Risk Difference | 95% CI | z | P | Weight (%) | |
|---|---|---|---|---|---|---|---|---|
| | | | | | | | Fixed | Random |
| IMpower150 | 3/393 | 0/394 | 0.00763 | -0.000972 to 0.0162 | | | 9.34 | 8.33 |
| IMpower133 | 2/198 | 0/196 | 0.0101 | -0.00383 to 0.0240 | | | 3.56 | 6.62 |
| IMpassion130 | 1/452 | 0/438 | 0.00221 | -0.00212 to 0.00654 | | | 36.85 | 9.45 |
| Keynote 407 | 4/278 | 4/280 | 0.000103 | -0.0196 to 0.0198 | | | 1.78 | 4.97 |
| KEYNOTE 189 | 13/405 | 0/202 | 0.0321 | 0.0149 to 0.0493 | | | 2.35 | 5.65 |
| NCT01285609 | 4/388 | 1/361 | 0.00754 | -0.00388 to 0.0190 | | | 5.30 | 7.43 |
| NCT02362594 | 4/509 | 1/502 | 0.00587 | -0.00274 to 0.0145 | | | 9.33 | 8.33 |
| keynote045 | 38/266 | 22/255 | 0.0566 | 0.00221 to 0.111 | | | 0.23 | 1.16 |
| Keynote024 | 2/154 | 0/150 | 0.0130 | -0.00489 to 0.0309 | | | 2.16 | 5.45 |
| Keynote 010 | 13/682 | 0/309 | 0.0191 | 0.00880 to 0.0293 | | | 6.56 | 7.80 |
| Pacific | 4/475 | 0/234 | 0.00842 | 0.000203 to 0.0166 | | | 10.24 | 8.45 |
| CheckMate 017 | 6/131 | 3/129 | 0.0225 | -0.0217 to 0.0668 | | | 0.35 | 1.65 |
| CheckMate 066 | 5/206 | 1/205 | 0.0194 | -0.00368 to 0.0425 | | | 1.30 | 4.19 |
| CheckMate 037 | 2/268 | 0/102 | 0.00746 | -0.00284 to 0.0178 | | | 6.51 | 7.79 |
| CheckMate 057 | 8/287 | 0/268 | 0.0279 | 0.00883 to 0.0469 | | | 1.91 | 5.14 |
| CheckMate 141 | 3/236 | 1/111 | 0.00370 | -0.0190 to 0.0264 | | | 1.35 | 4.28 |
| CA184-043 | 22/393 | 11/396 | 0.0282 | 0.000299 to 0.0561 | | | 0.89 | 3.31 |
| Total (fixed effects) | 134/5721 | 44/4532 | 0.0153 | 0.0105 to 0.0201 | 6.219 | <0.001 | 100.00 | 100.00 |
| Total (random effects) | 134/5721 | 44/4532 | 0.0122 | 0.00601 to 0.0185 | 3.852 | <0.001 | 100.00 | 100.00 |

**Test for heterogeneity**

| | |
|---|---|
| Q | 62.5155 |
| DF | 16 |
| Significance level | P < 0.0001 |
| $I^2$ (inconsistency) | 74.41% |
| 95% CI for $I^2$ | 58.81 to 84.10 |

**Figure S3.** G3-G4 renal toxicity.

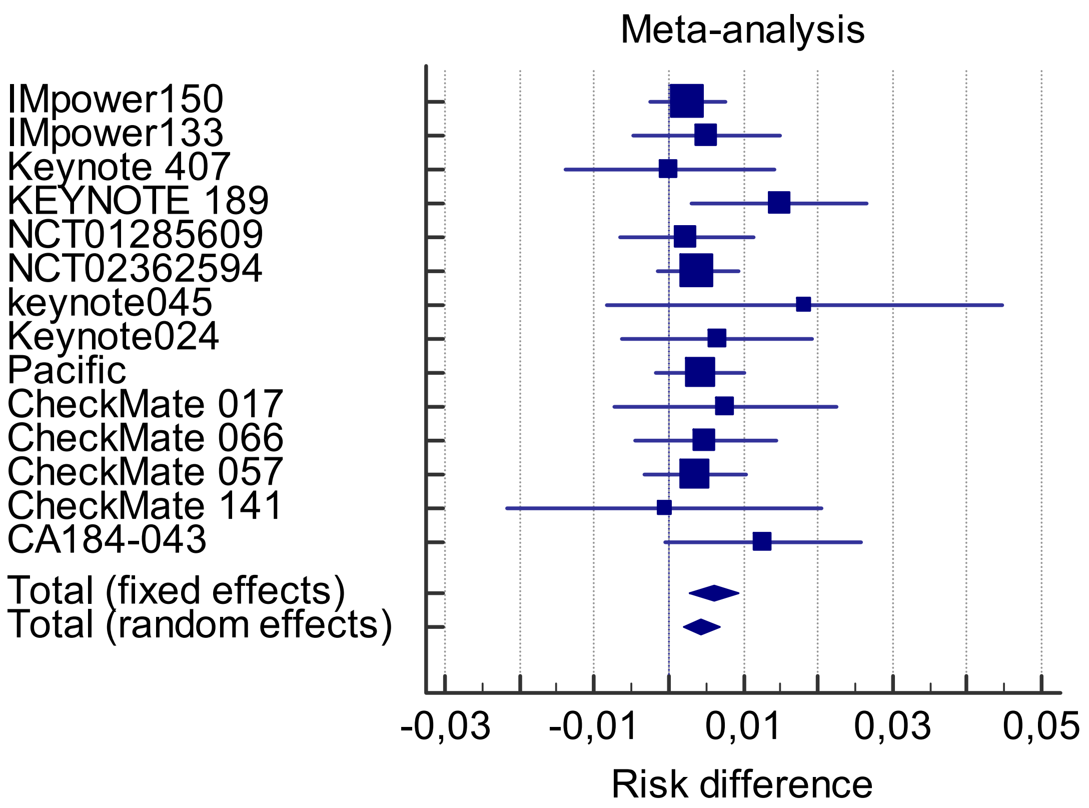

**Table S3.** G3-G4 renal toxicity.

| Study | Intervention | Controls | Risk Difference | 95% CI | z | P | Weight (%) | |
|---|---|---|---|---|---|---|---|---|
| | | | | | | | Fixed | Random |
| IMpower150 | 1/393 | 0/394 | 0.00254 | -0.00244 to 0.00753 | | | 21.04 | 21.04 |
| IMpower133 | 1/198 | 0/196 | 0.00505 | -0.00482 to 0.0149 | | | 5.36 | 5.36 |
| Keynote 407 | 2/278 | 2/280 | 0.0000514 | -0.0139 to 0.0141 | | | 2.66 | 2.66 |
| KEYNOTE 189 | 6/405 | 0/202 | 0.0148 | 0.00305 to 0.0266 | | | 3.77 | 3.77 |
| NCT01285609 | 2/388 | 1/361 | 0.00238 | -0.00657 to 0.0113 | | | 6.51 | 6.51 |
| NCT02362594 | 2/509 | 0/502 | 0.00393 | -0.00151 to 0.00936 | | | 17.67 | 17.67 |
| keynote045 | 9/266 | 4/255 | 0.0181 | -0.00840 to 0.0447 | | | 0.74 | 0.74 |
| Keynote024 | 1/154 | 0/150 | 0.00649 | -0.00619 to 0.0192 | | | 3.24 | 3.24 |
| Pacific | 2/475 | 0/234 | 0.00421 | -0.00161 to 0.0100 | | | 15.40 | 15.40 |
| CheckMate 017 | 1/131 | 0/129 | 0.00763 | -0.00727 to 0.0225 | | | 2.35 | 2.35 |
| CheckMate 066 | 1/206 | 0/205 | 0.00485 | -0.00464 to 0.0143 | | | 5.80 | 5.80 |
| CheckMate 057 | 1/287 | 0/268 | 0.00348 | -0.00333 to 0.0103 | | | 11.23 | 11.23 |
| CheckMate 141 | 2/236 | 1/111 | -0.000534 | -0.0216 to 0.0206 | | | 1.17 | 1.17 |
| CA184-043 | 6/393 | 1/396 | 0.0127 | -0.000350 to 0.0258 | | | 3.05 | 3.05 |
| Total (fixed effects) | 37/4319 | 9/3683 | 0.00610 | 0.00292 to 0.00929 | 3.755 | <0.001 | 100.00 | 100.00 |
| Total (random effects) | 37/4319 | 9/3683 | 0.00444 | 0.00216 to 0.00673 | 3.811 | <0.001 | 100.00 | 100.00 |

| | |
|---|---|
| Q | 9.3461 |
| DF | 13 |
| Significance level | P = 0.7463 |
| $I^2$ (inconsistency) | 0.00% |
| 95% CI for $I^2$ | 0.00 to 37.57 |

**Figure S4.** G1-G2 renal toxicity of PD-L1 and PD-1.

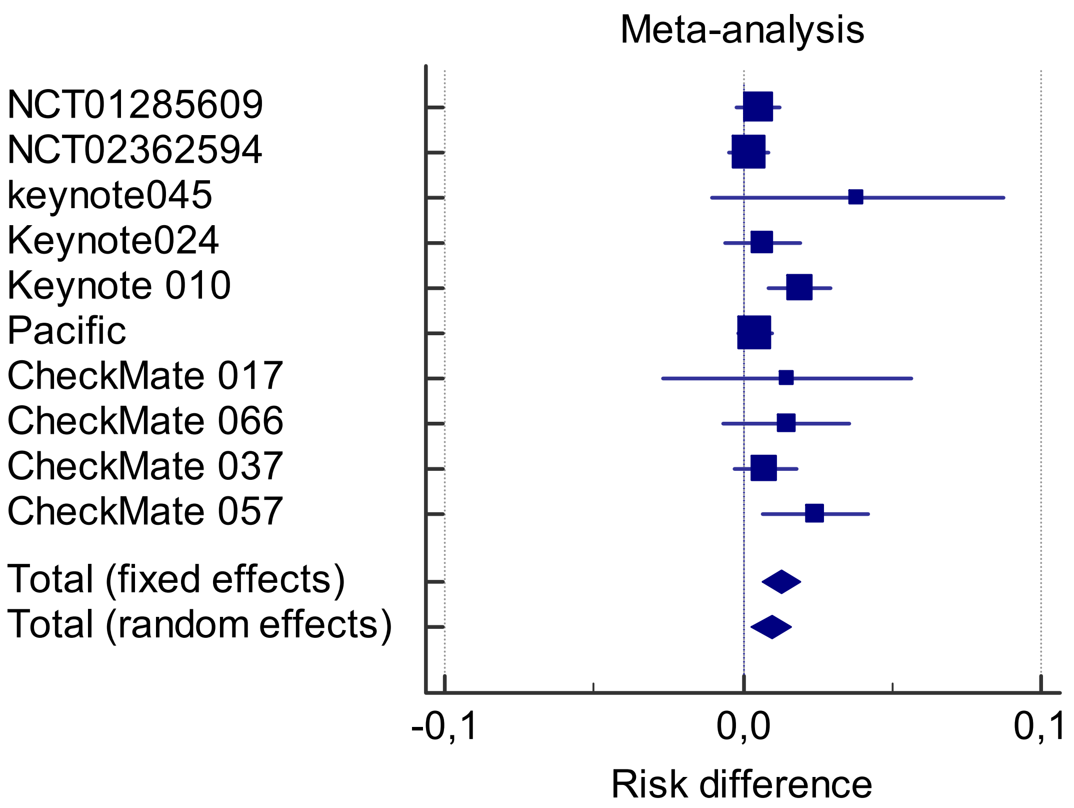

**Table S4.** G1-G2 renal toxicity of PD-L1 and PD-1.

| Study | Intervention | Controls | Risk Difference | 95% CI | z | P | Weight (%) | |
|---|---|---|---|---|---|---|---|---|
| | | | | | | | Fixed | Random |
| NCT01285609 | 2/388 | 0/361 | 0.00515 | -0.00197 to 0.0123 | | | 19.12 | 14.88 |
| NCT02362594 | 2/509 | 1/502 | 0.00194 | -0.00475 to 0.00863 | | | 21.69 | 15.19 |
| keynote045 | 29/266 | 18/255 | 0.0384 | -0.0105 to 0.0873 | | | 0.41 | 1.68 |
| Keynote024 | 1/154 | 0/150 | 0.00649 | -0.00619 to 0.0192 | | | 6.03 | 10.87 |
| Keynote 010 | 13/682 | 0/309 | 0.0191 | 0.00880 to 0.0293 | | | 9.22 | 12.58 |
| Pacific | 2/475 | 0/234 | 0.00421 | -0.00161 to 0.0100 | | | 28.62 | 15.78 |
| CheckMate 017 | 5/131 | 3/129 | 0.0149 | -0.0270 to 0.0568 | | | 0.55 | 2.22 |
| CheckMate 066 | 4/206 | 1/205 | 0.0145 | -0.00658 to 0.0357 | | | 2.18 | 6.40 |
| CheckMate 037 | 2/268 | 0/102 | 0.00746 | -0.00284 to 0.0178 | | | 9.14 | 12.55 |
| CheckMate 057 | 7/287 | 0/268 | 0.0244 | 0.00654 to 0.0422 | | | 3.05 | 7.84 |
| Total (fixed effects) | 67/3366 | 23/2515 | 0.0129 | 0.00692 to 0.0190 | 4.216 | <0.001 | 100.00 | 100.00 |
| Total (random effects) | 67/3366 | 23/2515 | 0.00959 | 0.00293 to 0.0162 | 2.821 | 0.005 | 100.00 | 100.00 |

**Test for heterogeneity**

| | |
|---|---|
| Q | 29.7132 |
| DF | 9 |
| Significance level | P = 0.0005 |
| $I^2$ (inconsistency) | 69.71% |
| 95% CI for $I^2$ | 41.81 to 84.23 |

**Figure S5.** G3-G4 renal toxicity of PD-L1 and PD-1.

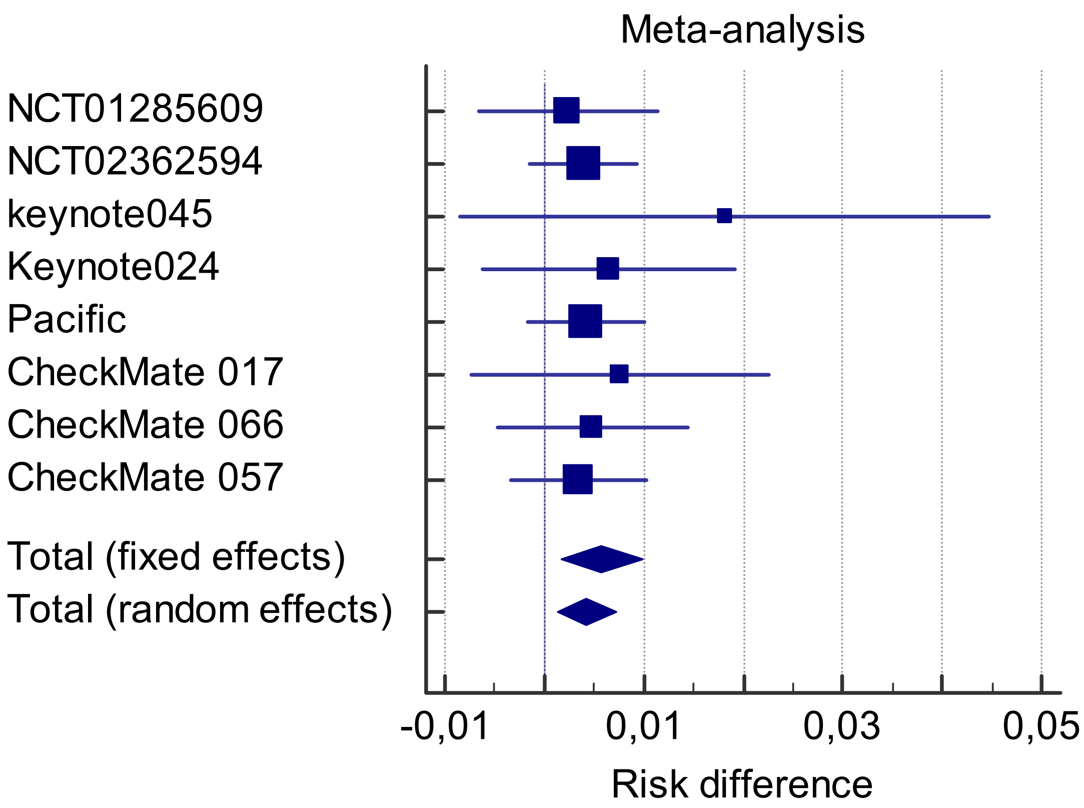

**Table S5.** G3-G4 renal toxicity of PD-L1 and PD-1.

| Study | Intervention | Controls | Risk Difference | 95% CI | z | P | Weight (%) | |
|---|---|---|---|---|---|---|---|---|
| | | | | | | | Fixed | Random |
| NCT01285609 | 2/388 | 1/361 | 0.00238 | -0.00657 to 0.0113 | | | 10.35 | 10.35 |
| NCT02362594 | 2/509 | 0/502 | 0.00393 | -0.00151 to 0.00936 | | | 28.08 | 28.08 |
| keynote045 | 9/266 | 4/255 | 0.0181 | -0.00840 to 0.0447 | | | 1.18 | 1.18 |
| Keynote024 | 1/154 | 0/150 | 0.00649 | -0.00619 to 0.0192 | | | 5.15 | 5.15 |
| Pacific | 2/475 | 0/234 | 0.00421 | -0.00161 to 0.0100 | | | 24.46 | 24.46 |
| CheckMate 017 | 1/131 | 0/129 | 0.00763 | -0.00727 to 0.0225 | | | 3.73 | 3.73 |
| CheckMate 066 | 1/206 | 0/205 | 0.00485 | -0.00464 to 0.0143 | | | 9.21 | 9.21 |
| CheckMate 057 | 1/287 | 0/268 | 0.00348 | -0.00333 to 0.0103 | | | 17.85 | 17.85 |
| Total (fixed effects) | 19/2416 | 5/2104 | 0.00580 | 0.00165 to 0.00995 | 2.740 | 0.006 | 100.00 | 100.00 |
| Total (random effects) | 19/2416 | 5/2104 | 0.00428 | 0.00140 to 0.00716 | 2.914 | 0.004 | 100.00 | 100.00 |

**Test for heterogeneity**

| | |
|---|---|
| Q | 2.6835 |
| DF | 7 |
| Significance level | P = 0.9127 |
| $I^2$ (inconsistency) | 0.00% |
| 95% CI for $I^2$ | 0.00 to 16.31 |

**Figure S6.** All grade renal toxicity of PD-L1 and PD-1.

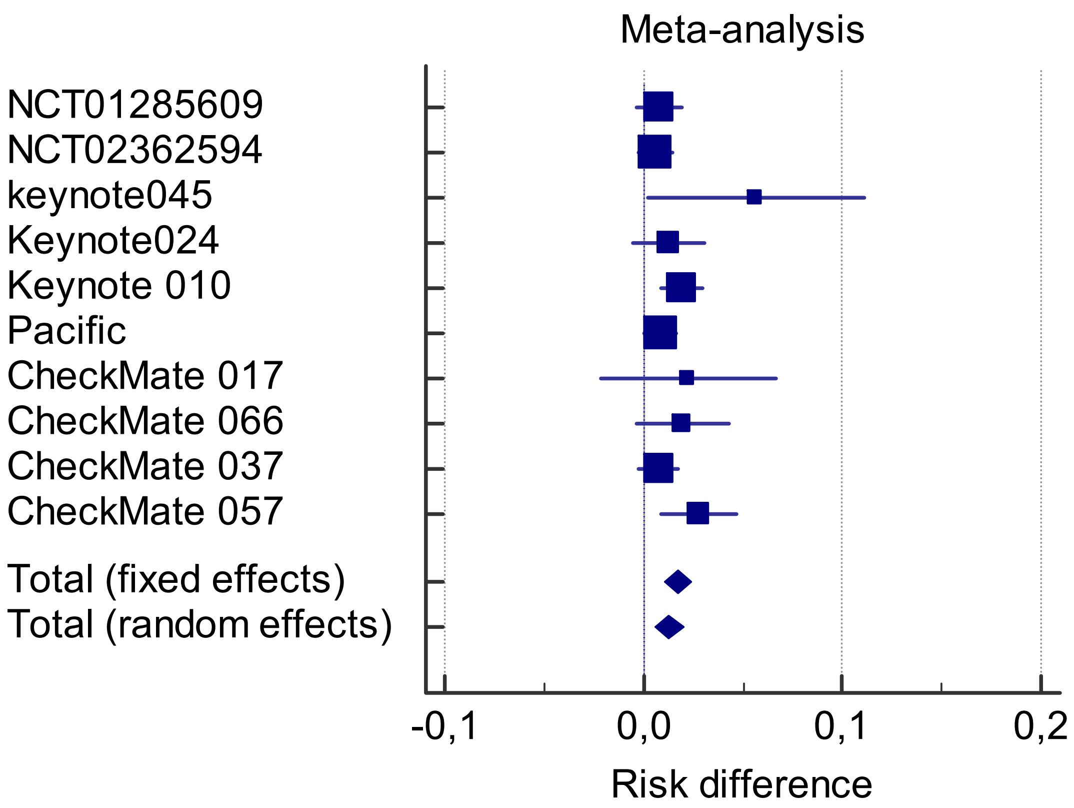

**Table S6.** All grade renal toxicity of PD-L1 and PD-1.

| Study | Intervention | Controls | Risk Difference | 95% CI | z | P | Weight (%) | |
|---|---|---|---|---|---|---|---|---|
| | | | | | | | Fixed | Random |
| NCT01285609 | 4/388 | 1/361 | 0.00754 | -0.00388 to 0.0190 | | | 12.07 | 13.21 |
| NCT02362594 | 4/509 | 1/502 | 0.00587 | -0.00274 to 0.0145 | | | 21.26 | 15.55 |
| keynote045 | 38/266 | 22/255 | 0.0566 | 0.00221 to 0.111 | | | 0.53 | 1.55 |
| Keynote024 | 2/154 | 0/150 | 0.0130 | -0.00489 to 0.0309 | | | 4.92 | 8.77 |
| Keynote 010 | 13/682 | 0/309 | 0.0191 | 0.00880 to 0.0293 | | | 14.95 | 14.16 |
| Pacific | 4/475 | 0/234 | 0.00842 | 0.000203 to 0.0166 | | | 23.32 | 15.87 |
| CheckMate 017 | 6/131 | 3/129 | 0.0225 | -0.0217 to 0.0668 | | | 0.80 | 2.25 |
| CheckMate 066 | 5/206 | 1/205 | 0.0194 | -0.00368 to 0.0425 | | | 2.96 | 6.37 |
| CheckMate 037 | 2/268 | 0/102 | 0.00746 | -0.00284 to 0.0178 | | | 14.83 | 14.12 |
| CheckMate 057 | 8/287 | 0/268 | 0.0279 | 0.00883 to 0.0469 | | | 4.34 | 8.15 |
| Total (fixed effects) | 86/3366 | 28/2515 | 0.0176 | 0.0108 to 0.0243 | 5.085 | <0.001 | 100.00 | 100.00 |
| Total (random effects) | 86/3366 | 28/2515 | 0.0130 | 0.00599 to 0.0201 | 3.629 | <0.001 | 100.00 | 100.00 |

**Test for heterogeneity**

| | |
|---|---|
| Q | 21.9713 |
| DF | 9 |
| Significance level | P = 0.0090 |
| $I^2$ (inconsistency) | 59.04% |
| 95% CI for $I^2$ | 17.67 to 79.62 |

**Figure S7.** G2-G3 renal toxicity of PD-1/PD-L1 combination.

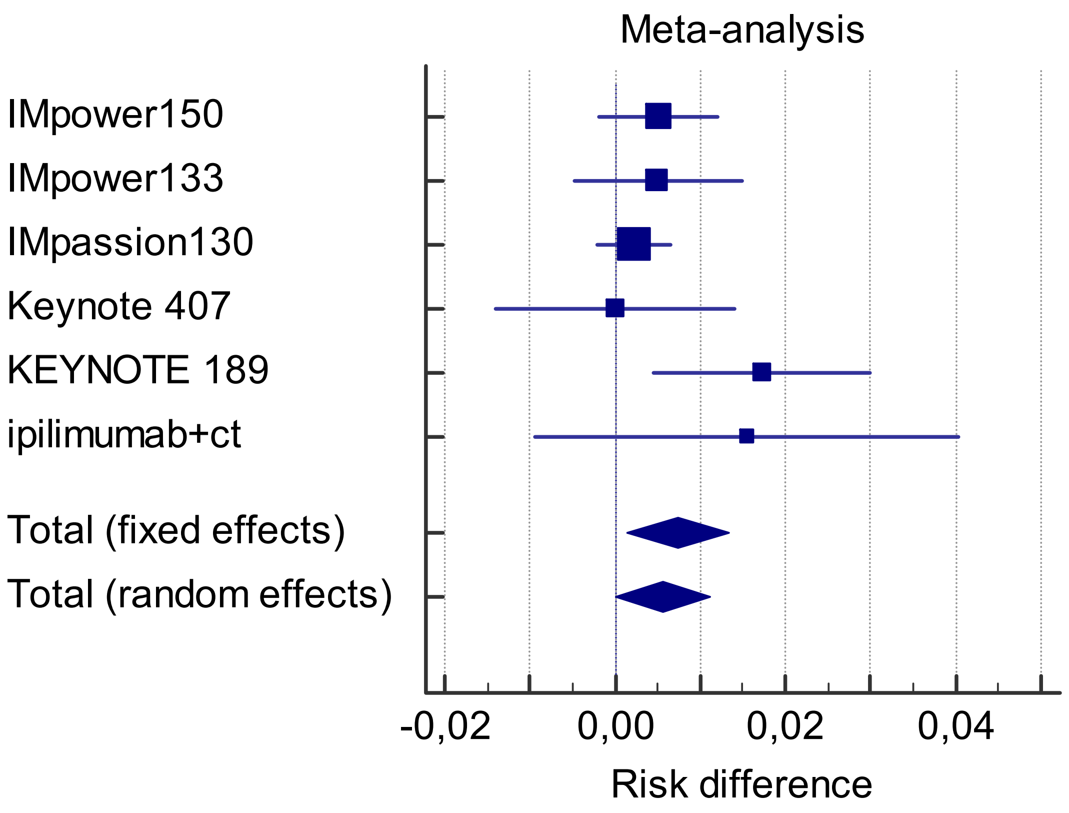

**Table S7.** G2-G3 renal toxicity of PD-1/PD-L1 combination.

| Study | Intervention | Controls | Risk Difference | 95% CI | z | P | Weight (%) | |
|---|---|---|---|---|---|---|---|---|
| | | | | | | | Fixed | Random |
| IMpower150 | 2/393 | 0/394 | 0.00509 | -0.00195 to 0.0121 | | | 20.90 | 23.58 |
| IMpower133 | 1/198 | 0/196 | 0.00505 | -0.00482 to 0.0149 | | | 10.61 | 17.30 |
| IMpassion130 | 1/452 | 0/438 | 0.00221 | -0.00212 to 0.00654 | | | 55.13 | 30.72 |
| Keynote 407 | 2/278 | 2/280 | 0.0000514 | -0.0139 to 0.0141 | | | 5.28 | 11.19 |
| KEYNOTE 189 | 7/405 | 0/202 | 0.0173 | 0.00459 to 0.0300 | | | 6.42 | 12.79 |
| ipilimumab+ct | 16/393 | 10/396 | 0.0155 | -0.00945 to 0.0404 | | | 1.67 | 4.43 |
| Total (fixed effects) | 29/2119 | 12/1906 | 0.00746 | 0.00150 to 0.0134 | 2.455 | 0.014 | 100.00 | 100.00 |
| Total (random effects) | 29/2119 | 12/1906 | 0.00565 | 0.0000706 to 0.0112 | 1.985 | 0.047 | 100.00 | 100.00 |

**Test for heterogeneity**

| | |
|---|---|
| Q | 10.0704 |
| DF | 5 |
| Significance level | P = 0.0733 |
| $I^2$ (inconsistency) | 50.35% |
| 95% CI for $I^2$ | 0.00 to 80.26 |

**Figure S8.** G3-G4 renal toxicity of PD-L1/PD-1 combination.

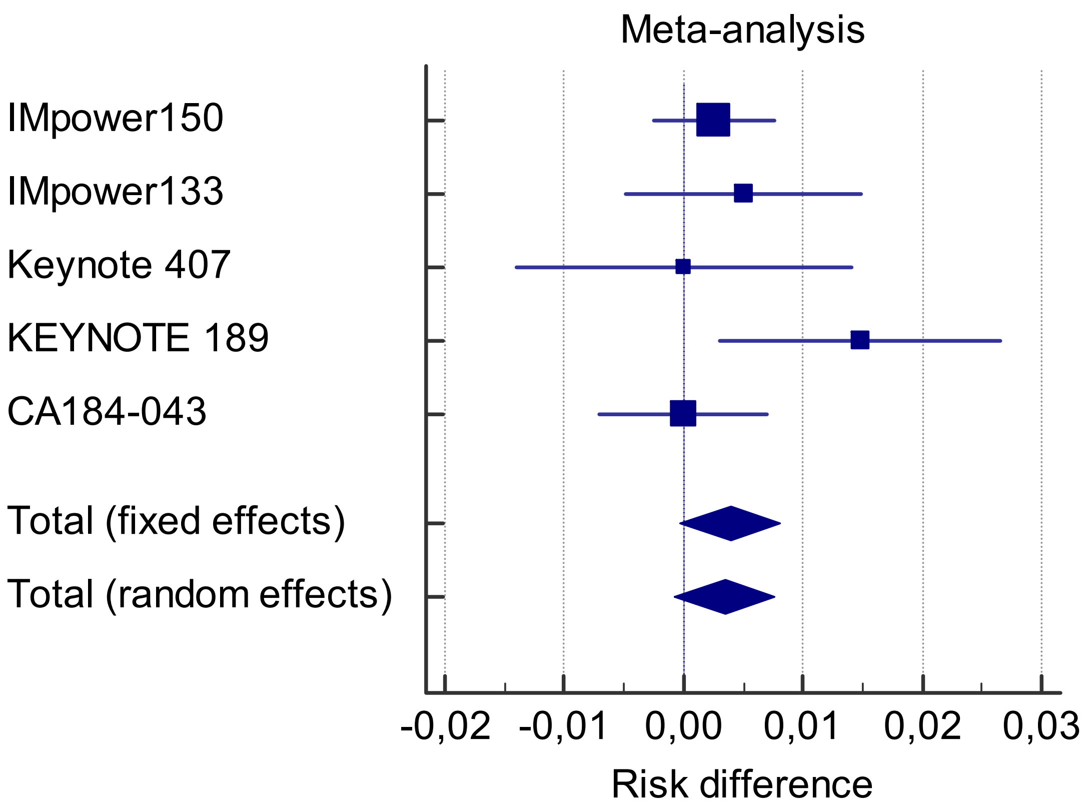

**Table S8.** G3-G4 renal toxicity of PD-L1/PD-1 combination.

| Study | Intervention | Controls | Risk Difference | 95% CI | z | P | Weight (%) | |
|---|---|---|---|---|---|---|---|---|
| | | | | | | | Fixed | Random |
| IMpower150 | 1/393 | 0/394 | 0.00254 | -0.00244 to 0.00753 | | | 48.45 | 39.77 |
| IMpower133 | 1/198 | 0/196 | 0.00505 | -0.00482 to 0.0149 | | | 12.33 | 15.12 |
| Keynote 407 | 2/278 | 2/280 | 0.0000514 | -0.0139 to 0.0141 | | | 6.13 | 8.22 |
| KEYNOTE 189 | 6/405 | 0/202 | 0.0148 | 0.00305 to 0.0266 | | | 8.68 | 11.21 |
| CA184-043 | 1/393 | 1/396 | 0.0000193 | -0.00700 to 0.00704 | | | 24.41 | 25.69 |
| Total (fixed effects) | 11/1667 | 3/1468 | 0.00392 | -0.000235 to 0.00808 | 1.849 | 0.064 | 100.00 | 100.00 |
| Total (random effects) | 11/1667 | 3/1468 | 0.00344 | -0.000766 to 0.00766 | 1.604 | 0.109 | 100.00 | 100.00 |

**Test for heterogeneity**

| | |
|---|---|
| Q | 5.1173 |
| DF | 4 |
| Significance level | P = 0.2755 |
| $I^2$ (inconsistency) | 21.83% |
| 95% CI for $I^2$ | 0.00 to 67.33 |

**Figure S9.** All grade renal toxicity of PD-L1/PD-1 combination.

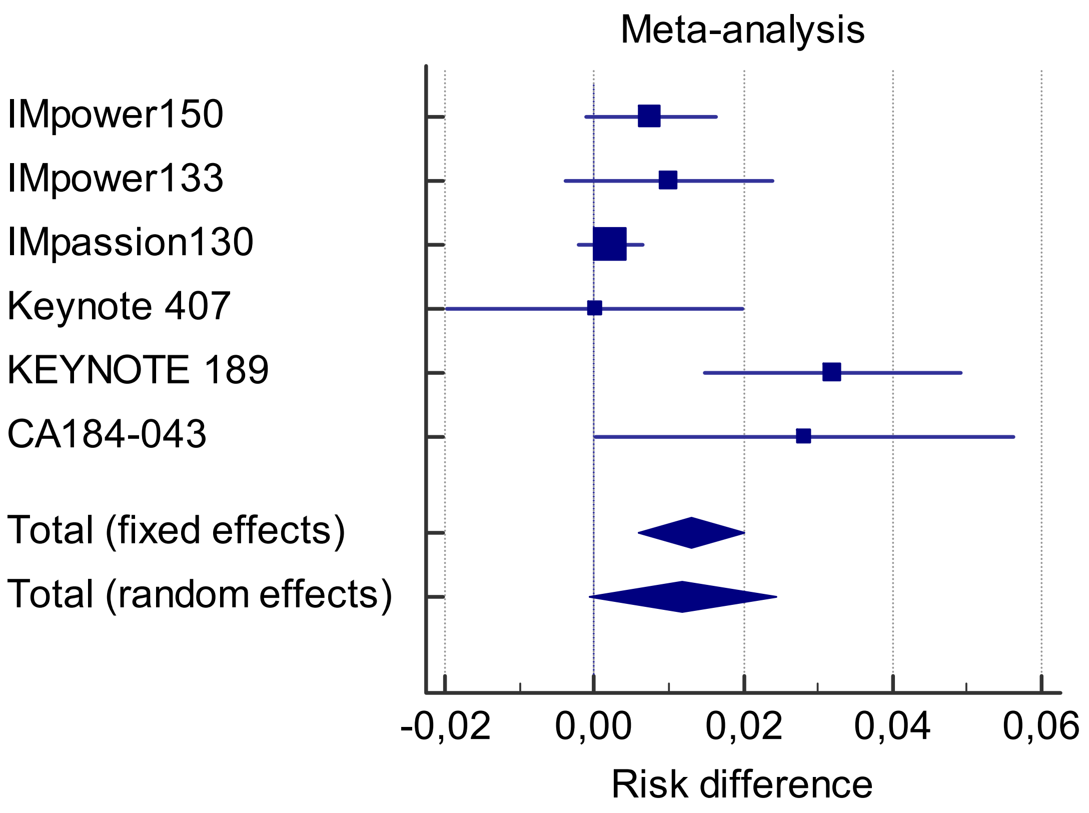

**Table S9.** All grade renal toxicity of PD-L1/PD-1 combination.

| Study | Intervention | Controls | Risk Difference | 95% CI | z | P | Weight (%) | |
|---|---|---|---|---|---|---|---|---|
| | | | | | | | Fixed | Random |
| IMpower150 | 3/393 | 0/394 | 0.00763 | -0.000972 to 0.0162 | | | 17.05 | 20.19 |
| IMpower133 | 2/198 | 0/196 | 0.0101 | -0.00383 to 0.0240 | | | 6.51 | 17.47 |
| IMpassion130 | 1/452 | 0/438 | 0.00221 | -0.00212 to 0.00654 | | | 67.29 | 21.75 |
| Keynote 407 | 4/278 | 4/280 | 0.000103 | -0.0196 to 0.0198 | | | 3.24 | 14.33 |
| KEYNOTE 189 | 13/405 | 0/202 | 0.0321 | 0.0149 to 0.0493 | | | 4.28 | 15.70 |
| CA184-043 | 22/393 | 11/396 | 0.0282 | 0.000299 to 0.0561 | | | 1.62 | 10.55 |
| Total (fixed effects) | 45/2119 | 15/1906 | 0.0130 | 0.00596 to 0.0201 | 3.614 | <0.001 | 100.00 | 100.00 |
| Total (random effects) | 45/2119 | 15/1906 | 0.0118 | -0.000658 to 0.0243 | 1.857 | 0.063 | 100.00 | 100.00 |

**Test for heterogeneity**

| | |
|---|---|
| Q | 33.1801 |
| DF | 5 |
| Significance level | P < 0.0001 |
| $I^2$ (inconsistency) | 84.93% |
| 95% CI for $I^2$ | 69.02 to 92.67 |

**Table S10.** G1-G2 renal toxicity of CTLA4.

| Study | Intervention | Controls | Risk Difference | 95% CI | z | P | Weight (%) | |
|---|---|---|---|---|---|---|---|---|
| | | | | | | | Fixed | Random |
| CheckMate 141 | 1/236 | 0/111 | 0.00424 | -0.00405 to 0.0125 | | | 90.04 | 60.69 |
| CA184-043 | 16/393 | 10/396 | 0.0155 | -0.00945 to 0.0404 | | | 9.96 | 39.31 |
| Total (fixed effects) | 17/629 | 10/507 | 0.0124 | -0.00581 to 0.0305 | 1.333 | 0.182 | 100.00 | 100.00 |
| Total (random effects) | 17/629 | 10/507 | 0.00865 | -0.0162 to 0.0335 | 0.683 | 0.494 | 100.00 | 100.00 |

**Test for heterogeneity**

| | |
|---|---|
| Q | 3.7443 |
| DF | 1 |
| Significance level | P = 0.0530 |
| $I^2$ (inconsistency) | 73.29% |
| 95% CI for $I^2$ | 0.00 to 93.98 |

**Table S11.** G3-G4 renal toxicity of CTLA4.

| Study | Intervention | Controls | Risk Difference | 95% CI | z | P | Weight (%) | |
|---|---|---|---|---|---|---|---|---|
| | | | | | | | Fixed | Random |
| CheckMate 141 | 2/236 | 1/111 | -0.000534 | -0.0216 to 0.0206 | | | 27.77 | 29.74 |
| CA184-043 | 6/393 | 1/396 | 0.0127 | -0.000350 to 0.0258 | | | 72.23 | 70.26 |
| Total (fixed effects) | 8/629 | 2/507 | 0.00907 | -0.00206 to 0.0202 | 1.597 | 0.110 | 100.00 | 100.00 |
| Total (random effects) | 8/629 | 2/507 | 0.00879 | -0.00310 to 0207 | 1.449 | 0.147 | 100.00 | 100.00 |

**Test for heterogeneity**0.

| | |
|---|---|
| Q | 1.0972 |
| DF | 1 |
| Significance level | P = 0.2949 |
| $I^2$ (inconsistency) | 8.86% |
| 95% CI for $I^2$ | 8.86 to 8.86 |

**Table S12.** All grades renal toxicity of CTLA4.

| Study | Intervention | Controls | Risk Difference | 95% CI | z | P | Weight (%) | |
|---|---|---|---|---|---|---|---|---|
| | | | | | | | Fixed | Random |
| CheckMate 141 | 3/236 | 1/111 | 0.00370 | -0.0190 to 0.0264 | | | 60.27 | 53.99 |
| CA184-043 | 22/393 | 11/396 | 0.0282 | 0.000299 to 0.0561 | | | 39.73 | 46.01 |
| Total (fixed effects) | 25/629 | 12/507 | 0.0214 | 0.000289 to 0.0426 | 1.987 | 0.047 | 100.00 | 100.00 |
| Total (random effects) | 25/629 | 12/507 | 0.0150 | -0.0138 to 0.0437 | 1.021 | 0.307 | 100.00 | 100.00 |

**Test for heterogeneity**

| | |
|---|---|
| Q | 2.5764 |
| DF | 1 |
| Significance level | P = 0.1085 |
| $I^2$ (inconsistency) | 61.19% |
| 95% CI for $I^2$ | 0.00 to 90.99 |
